# Raring to go? A cross-sectional survey of student paramedics on how well they perceive their UK pre-registration course to be preparing them to manage suspected seizures

**DOI:** 10.1186/s12873-023-00889-5

**Published:** 2023-10-08

**Authors:** Adam J. Noble, Carolyn Lees, Kay Hughes, Lucy Almond, Hesham Ibrahim, Cerys Broadbent, Pete Dixon, Anthony G. Marson

**Affiliations:** 1 Department of Public Health, Policy and Systems, University of Liverpool, Ground Floor, Whelan Building, Liverpool, L69 3GL UK; 2 School of Health Sciences, University of Liverpool, Liverpool, UK; 3 School of Public and Allied Health, Liverpool John Moores University, Liverpool, UK; 4 Department of Psychology, University of Liverpool, Liverpool, UK; 5 Department of Primary Care and Mental Health, University of Liverpool, Liverpool, UK; 6 Department of Pharmacology and Therapeutics, University of Liverpool, Liverpool, UK

**Keywords:** Paramedics, Emergency medical services, Ambulance, Seizures, Epilepsy, Students, Decision making

## Abstract

**Background:**

Paramedics convey a high proportion of seizure patients with no clinical need to emergency departments (EDs). In a landmark study, only 27% of UK paramedics reported being “Very…”/ “Extremely confident” making seizure conveyance decisions. Improved pre-registration education on seizures for paramedics is proposed. Clarity is needed on its potential given recent changes to how UK paramedics train (namely, degree, rather than brief vocational course). This study sought to describe UK student paramedics’ perceived readiness to manage seizures and educational needs; compare this to what they report for other presentations; and, explore subgroup differences.

**Methods:**

Six hundred thirty-eight students, in year 2 or beyond of their pre-registration programme completed a cross-sectional survey. They rated perceived confidence, knowledge, ability to care for, and educational needs for seizures, breathing problems and, headache. Primary measure was conveyance decision confidence.

**Results:**

For seizures, 45.3% (95% CI 41.4–49.2) said they were “Very…”/“Extremely confident” to make conveyance decisions. This was similar to breathing problems, but higher than for headache (25.9%, 95% CI 22.6–29.5). Two hundred and thirty-nine participants (37.9%, 95% CI 34.1–41.8) said more seizure education was required – lower than for headache, but higher than for breathing problems. Subgroup differences included students on university-based programmes reporting more confidence for conveyance decisions than those completing degree level apprenticeships.

**Conclusions:**

Student paramedics report relatively high perceived readiness for managing seizures. Magnitude of benefit from enhancements to pre-registration education may be more limited than anticipated. Additional factors need attention if a sizeable reduction to unnecessary conveyances for seizures is to happen.

**Supplementary Information:**

The online version contains supplementary material available at 10.1186/s12873-023-00889-5.

## Background

### Seizures and the ambulance service

Each year, England’s ambulance services respond to ~ 211,000 calls for suspected seizures – making them the seventh most common presentation [1, 2]. The care offered should align with patient need and represent efficient resource use. This may not always be happening. Data indicates adults have often been taken by paramedics to emergency departments (EDs), despite no clinical need [1, 3–5]. Other countries report similar issues [6, 7].

### Need for conveyance to emergency department after a seizure

Seizures can be complex as they can be symptomatic of a wide range of brain pathologies, and ED for them can be important. Reasons ED might be required include status epileptics, a seizure in the context of known or possible pregnancy, significant actual or potential injury and persistent changes in awareness or behaviour that may jeopardize safety if left alone. However, as noted by ambulance care guidelines [8], for most adult cases seen by paramedics, ED is not required. Many occur in the context of an established diagnosis, such as epilepsy or functional neurological disorder [4, 9], and present little need for ED. Dickson et al. [1] reviewed records from one English ambulance service. Seizures had self-terminated in > 90% of cases, breathing was normal for > 96% and most people were recovering without intervention. Nevertheless, crews advised ED for 89%.

Taking a person to ED who does not require it, results in a ‘avoidable attendance’ (AA). As well as being potentially inconvenient [10], AAs can harm the patient due to unnecessary investigations/treatments [11], and have implications for others since they restrict ED capacity [12]. They are also costly [13].

### Insufficient education on seizures for paramedics identified as important

So, what can be done to reduce AAs for seizures? One suggestion is improved seizure education for paramedics [14]. This is because studies with paramedics in the United Kingdom (UK) [15–18] consistently indicate many believe their education on seizures was insufficient, meaning their knowledge of the presentation can be inadequate and they feel apprehensive about managing them. Key quotes from qualitative studies with practicing UK paramedics on this topic include:

*“Training on managing seizures…you might get a couple of hours, if that… The focus is really on the emergency side of things.”* [16]

*“We’re really good at dealing with respiratory disorders and we’re really good at dealing with heart attacks. We’ve had so much focus on those conditions … I just don’t think that neurological disorders people feel the same level of confidence generally”* [17]

*“There is this…sort of anxiety…the patient presentation is slightly beyond what you’re comfortable with [so] you take the patient to ED…”* [15]

*“I don’t mind sitting there for an hour or so just trying to convince them [the patient] to go to hospital”* [15]

Non-emergency states, such as terminated or self-resolving seizures, are described as particularly difficult to manage, with paramedics saying they often have little confidence in identifying the needs of patients and deciding whether ED conveyance is necessary. Indeed, Kinney et al. [18] surveyed UK ambulance clinicians and found only a minority were confident in making conveyance decisions.

### Is paramedic education *still* insufficient?

It is unclear whether pre-registration education still requires improvement. Why? Because the earlier findings come from studies whose samples were dominated by paramedics trained via the traditional, vocational system. Those now entering services have, in contrast, qualified via a higher education-supported model.

Rather than completing a 6–8 week theoretical programme with a period of consolidation in practice, UK trained paramedics now complete a professional regulator approved [19], 3–4 year university-based degree programme or a 2–4 year degree level apprenticeship. Since ~ 2021 only paramedic educational programmes at or above degree level have been permitted to admit new learners.

### Current study

Any change to paramedic education requires careful justification. A systematic search of the literature found no evidence on how well the higher education-supported model is preparing paramedics to manage seizures (Additional file 1). Therefore, this study reports a survey of current UK student paramedics. It sought to:

1. Describe their perceived readiness to manage seizures and educational needs;
2. Compare this to what they reported for some other patient presentations; and
3. Explore whether perceived readiness was related to specific type of education or academic year of study.

## Methods

### Design

An anonymous cross-sectional online “open” survey hosted by Qualtrics was conducted between November 2022 and January 2023. Developed in consultation with stakeholders, it asked student paramedics about their perceived confidence, knowledge, ability to care for, and educational needs for, persons experiencing three different presentations. They were seizures, breathing problems and, headache. We followed Porter [20] in understanding self-confidence as the belief in one’s ability to accomplish a specific goal or task [21] and closely related to Bandura’s [22] concept of self-efficacy. Whilst actual knowledge refers to possession of information involved in seizure management, we, in line with Park et al. [23], understood perceived knowledge as referring to one’s self-assessment or feeling of knowing the information needed for seizure management.

To avoid providing explicit cues as to the study’s aims and influencing responses, the survey’s interest in seizures was obscured.

### Eligibility criteria

To participate, respondents needed to confirm they were aged ≥ 16 years; enrolled on a UK educational programme that would qualify them for paramedic registration; were in year 2 or beyond; and that they could independently complete a questionnaire in English. Educators advised restricting participation to students in year 2 or beyond because the ‘spiral’ framework followed by paramedic programmes [19] meant it unlikely seizures would have been considered in detail until this point.

### Recruitment

Thirty-one (67.4%) of the 46 universities offering ≥ 1 approved [24] paramedic courses at the time and who had students in years 2 or beyond cascaded a recruitment advert to students (Additional file 2 and Acknowledgements). The advert was also posted within social media groups for student paramedics (Acknowledgements). Interested persons were directed to a survey page. Participation was voluntary. To incentivise recruitment, the first 300 people submitting complete responses each received a £5 voucher.

### Ethical approval

Approval was provided by the University of Liverpool’s Ethics Committee (Ref: 11962). All participants provided informed consent and could download a Participant Information Sheet that informed them of the survey's length, which data were stored and where and for how long, who the investigators were, and the purpose of the study. Reporting conforms with the CHERRIES statement.

### Survey content

#### Overview

After questions about their characteristics, participants were presented with a series of measures to complete for each presentation. The order the presentations were asked about was randomised by Qualtrics. Additional file 3 provides the full survey.

The number of questionnaire pages was 21, with items per page ranging from 1 to 6. ‘Responsive question’ techniques were used to minimise burden and validation was used to force respondents to answer all applicable questions. No ‘back button’ or review step was incorporated into the survey to enable participants to change their answers. No procedures were used to prevent or screen out multiple submissions. Search engines were though, blocked from including the survey in their search results.

Breathing problems and headache were considered informative comparators. Breathing problems are frequently seen by paramedics (third most common) [1, 2], but are less likely to be unnecessarily conveyed [3]. Headache in contrast is infrequently seen by paramedics (twenty fourth most seen presentation) [1, 2], but it has a high rate of AA following ambulance attendance [3].

#### Measures

For each presentation, participants were administered the following:

##### Confidence in making conveyance decisions

Participants were asked “How confident would you say you would be in deciding whether or not to convey a ‘X’ patient to ED?”. As per Kinney et al.’s [18] study, participants responded using a 5-point Likert scale (1 = “Not at all confident”; 2 = “Slightly confident”; 3 = “Reasonably confident”; 4 = “Very confident”; 5 = “Extremely confident”).

##### Perceived knowledge of, ability to care for and confidence to care

Waltrich’s [25] 15-item questionnaire, with minor adjustments, was used. It asked participants to rate perceived knowledge (e.g., “My knowledge of ‘X’ patients is comprehensive”), ability to provide care (e.g., “I believe my education and training is preparing me well to provide care that benefits ‘X’ patients”) and confidence to care (e.g.,” ‘I would feel comfortable in my ability if I were to attend a patient with a ‘X’ problem’). They responded using a 5-point Likert scale (1 = “Strongly disagree”, 5 = “Strongly agree”); some items were reverse scored.

As the measure was designed for use with qualified paramedics, we amended it to remove references to the person’s everyday practice to make it suitable for students (Additional file 4).

Participants responses to the items were totalled and, as per the measure’s manual, converted into a percentage of maximum possible (POMP) score (range 0–100, higher scores indicating higher perceived ability).

The scale’s internal consistency was acceptable (α range 0.75 to 0.85).

##### Educational need

Participants were asked “Do you think you should/should have received more training on ‘X’ via your pre-registration programme?” Response options were Yes, Unsure and No.

### Analyses

#### Sample size

Participants’ confidence to make conveyance decisions for seizures was the primary parameter our descriptive study sought to estimate – specifically the proportion who perceived themselves to “Very…”/ “Extremely confident”. To calculate the required sample size, the following formula was used: N = (P(100%-P))/(SE)^2^, where P is the anticipated proportion and SE the standard error.

No existing estimate was available on the anticipated proportion of student paramedics that would be found to report being “Very…”/ “Extremely confident”. However, in Kinney et al.’s [18] study, 27.7% *practicing* paramedics did report this. This was used as P for the calculations. We stipulated a need for the sample to be sufficient to mean there would be 99% confidence that the estimate generated was within ± 5% of the true proportion – thus the SE was 1.95 (5/2.56). Using these figures, the sample size calculation stated a need for 527 participants with complete data.

#### Statistical analyses

Analyses were completed using data from participants with valid submissions – defined as a participant having, as a minimum, completed Waltrich’s [25] measure (it appeared first in our survey pack, after the demographic questions). No imputation occurred. Where available, the characteristics of individuals who did and did not make a valid submission were presented side-by-side to help evaluate representativeness.

The data from the 3 measures differed in nature and so different tests were required. The tests used for the different measures and the reasons why were as follows:

- *Confidence in making conveyance decisions:* Given the restricted number of ordinal categories available to respond to the conveyance confidence question, central tendency is described according to the median (Mdn) and interquartile range (IQR). Friedman’s 2-way ANOVA compared participants’ responses for the three presentations. Mann–Whitney U tests explored whether responses differed according to education type (university-based vs apprenticeship) or year of study (year 2 or later).
- *Perceived knowledge of, ability to care for and confidence to care:* For Waltrich’s [25] questionnaire, there was a higher number of occupied categories and the distribution for POMP scores approximated normal. Therefore, means (M) and standard deviations (SDs) are used. Repeated measures one-way ANOVAs compared participants’ scores for the presentations. Independent t-tests (with bootstrapping) explored subgroup differences (education type; year of study).
- *Educational need:* The proportion of participants saying “Yes” more training for seizures was required is reported. Cochran’s Q Test compared the proportions saying this for the different presentations, whilst the Chi-square test explored subgroup differences (education type; year of study).

To account for multiple comparisons, alpha for all main analyses was set at 0.01. Only statistically significant subgroup differences are reported.

## Results

### Responses

There were *n* = 685 survey submissions. Of these, *n* = 638 (93.1%) were valid. It took them a mean of 23.4 min to complete the survey (SD 8.9).

Those who started, but did not sufficiently complete the measures for it to be considered valid were broadly comparable to those who did (Table 1).

**Table 1:** Characteristics of student paramedics with valid submissions for inclusion in data-set

	**Included in data-set**
**Characteristic**	***N* = 638**
***Age**** Median (interquartile range)*	23 (20, 26)
Missing	0
***Sex,**** n (%)*	
Male	174 (27.3%)
Female	464 (72.7%)
Prefer not to say	0
Missing	0
***Training route,**** n (%)*	
University-based (BSc, MSc)^a^	506 (79.3%)
Apprenticeship	132 (20.7)
Missing	0
***Year of current study,**** n (%)*	
Year 2	262 (41.1%)
Year 3	312 (48.9%)
Year 4	64 (10.0%)
Missing	0
***Location of training within UK,**** n (%)*	
Northern Ireland	5 (0.8%)
Scotland	69 (10.8%)
Wales	21 (3.3%)
England	543 (85.1%)
Missing	0
North West	93 (17.1%)
West Midlands	90 (16.6%)
Yorkshire and Humber	84 (15.5%)
South West	77 (14.2%)
South East	67 (12.3%)
East of England	54 (9.9%)
London	45 (8.3%)
East Midlands	33 (6.1%)

*BSc* Bachelor of Science, *MSc* Master of Science

^a^includes nurse paramedic course

### Participant characteristics

The median age of the *n* = 638 participants was 23 (IQR 20–26), with 464 (72.7%) being female (Table 1). Most (85.1%) were students in England and university-based (79.3%), rather than studying via an apprenticeship (21.4%).

Participants who were university-based and those on an apprenticeship were similar in age and sex. Those on an apprenticeship were less likely to be in year 2 (21.2 vs 46.1%; *X*^*2*^(1) = 26.662, *p* < 0.001).

### Measures

#### Confidence in making conveyance decisions

With respect to seizures, 45.3% (95% Confidence Interval [CI] 41.4–49.2) of participants said they perceived themselves to be “Very…” or “Extremely confident” to make non-conveyance decisions (Table 2).

**Table 2:** Confidence *n* = 638 participants reported to make conveyance decisions by presentation

***Confidence in making conveyance decisions measure***	***Presentation***		
	*Seizure*	*Breathing problem*	*Headache*
*Extremely confident, n %*	107 (16.8%)	105 (16.5%)	54 (8.5%)
*Very confident, n %*	181 (28.5%)	215 (33.8%)	111 (17.5%)
*Reasonably confident, n %*	212 (33.3%)	188 (29.6%)	229 (36.0%)
*Slightly confident, n %*	97 (15.3%)	97 (15.3%)	175 (27.5%)
*Not at all confident, n %*	39 (6.1%)	31 (4.9%)	67 (10.5%)
Missing	2	2	2

*IQR* interquartile range, *n* number

For breathing problems, 50.3% (95% CI 46.4–54.3) said they were “Very…” or “Extremely confident”, whilst for headache it was 25.9% (95% CI 22.6–29.5). In line with this, perceived confidence differed significantly for the presentations (Friedman χ2 = 125.599 (2), *p* < 0.001). Bonferroni comparisons found confidence for headache to be significantly lower than for seizures ( *r* = -0.23) and breathing problems (*r* = -0.26).

A subgroup difference was that those on a university-based course expressed significantly more confidence for seizures than those studying via an apprenticeship (*U* = 25,612.0, *p* < 0.01; *r* = 0.16); 49.2% of the former described themselves as “Very…” or “Extremely confident” compared to 30.3% of the latter. They also expressed more confidence for breathing problems (*U* = 26,985.0, *p* < 0.01; *r* = 0.14).

#### Perceived knowledge of, ability to care for and confidence to care

The mean POMP score for seizures was 66.6 (95% CI 63.7–65.0; SD 11.3).

POMP scores for the different presentations varied significantly (*F*(1.95, 1274) = 152.046, *p* < 0,001; *ɛ* = 0.97; η_p_^2^ = 0.19). Participants expressed lower scores for seizures (mean difference[MD] = -6.5, 95% CI -7.9 to -5.1) and headaches (MD = -8.6, 95% CI -10.3 to -6.9) than for breathing problems.

The POMP score for headaches was also significantly lower than for seizures (mean difference = -2.1, 95% -3.6 to -0.6).

Compared to those studying via an apprenticeship, those who were university-based reported significantly higher POMP scores for seizures (MD = 4.4, bias-corrected and accelerated bootstrap [BCa] 99% CI 2.2–6.8; *d* = 0.38) and breathing problems (MD = 11.5, BCa 99% CI 8.9–14.1; *d* = 0.88). Those in year 3 or beyond of their studies had significantly higher POMP score for breathing problems compared to those in year 2 (MD = 3.1, BCa 99% CI 0.4–5.7; *d* = 0.23).

#### Educational need

Two hundred and thirty-nine participants (37.9%, 95% CI 34.1–41.8) said “Yes” more training was required for seizures.

**Fig. 1:**
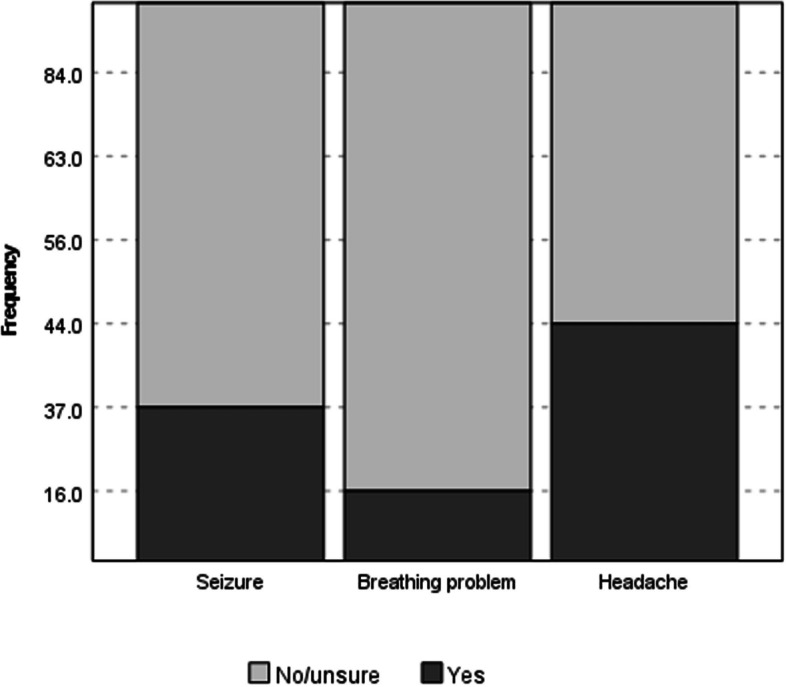
Proportion of participants stating “yes” more pre-registration education was required for the different presentations

The proportion saying more was required differed significantly for the presentations (χ^2^(2) 171.750, *p* < 0.001). Specifically, more expressed a need for training on headache (44.5%, 95% CI 40.6–48.5) than for seizures or breathing problems (17.0%, 95% CI 14.1–20.1). The proportion wanting more on seizures was also higher than for breathing problems (Fig. 1).

Those in year 2 were more likely to identify a need for more on seizures (44.2% vs 33.7% *X*^2^ = 7.141, *p* = 0.008) than those in year 3 or beyond.

## Discussion

### Main findings

Interventions to reduce AAs for seizures are sought. We undertook what is, to our knowledge, the largest survey of UK student paramedics to understand the potential utility of enhancements to pre-registration education for paramedics.

Results suggest the magnitude of benefit may be lower than anticipated. Whilst studies with practising paramedics signal low confidence for managing seizures [15–18], our student participants expressed high confidence. Conveyance decisions are a case in point – 45% of students rated themselves as being extremely/very confident. Only 27% of practising ambulance clinicians said this when asked by Kinney [18].

What was notable was the confidence students reported was not dissimilar to that which they expressed for breathing problems – a presentation frequently seen by paramedics [1, 2], for which conveyance decisions are known to reasonably accurate [3]. Students’ confidence for managing seizures was also higher than for headaches. As might be anticipated [19], confidence for seizures was higher for those later on in their training.

So, what might explain the high confidence of students? It could be due to the professionalisation of paramedics and the recent shift in their education from a largely vocational, short model of training to a longer, higher education model. Reasons for the shift included concerns that the vocational model fostered focused on lifesaving conditions and that there was a need for paramedics to be more autonomous and able to decide whether patients can be assessed and treated in their own homes or require transport to hospital [26]. Our findings may suggest the move is achieving its goal. Previous studies have suggested differences in the decision making [27, 28] of those qualifying by the vocational and higher education routes.

An alternative explanation relates to how students’ readiness was determined. They self-assessed it. Whilst practical and widely used, it remains unclear how accurately people, including student paramedics [28, 29], can assess their abilities. Evidence from the wider literature, such as on the Dunning-Kruger cognitive bias [30], indicates those who perform best on some objective assessment may underestimate their performance, while lower performers can overestimate it. Also of potential relevance is the ‘theory–practice’ gap [31]. Specifically, when trainees qualify and seek to apply their theoretical knowledge to the complexity of the workplace and make decisions their confidence may diminish. We did not ask participants what clinical exposure they had with seizures. A previous report noted students spend ~ 1625 h on clinical practice hours over a 3-year programme [32]. What they encounter will though, vary. Future studies should explore what, if any relevance, such phenomenon have.

### Implications

If participants’ perceived readiness reflects actual readiness, then approximately a third of our participants called for further pre-registration education on seizures. It remains unclear whether the expressed need justifies a change to pre-registration provision. This is a judgement that the wider community and stakeholders need to take a view on. It is possible that some of the need might be addressed by the time participants have completed more of their existing pre-registration programme.

Another issue that stakeholders should consider is the lower sense of readiness for managing seizures reported by those training via an apprenticeship compared to those who were university-based. To our knowledge, this is the first comparison of students from the different pathways. Why the difference exists is unknown. Might it be due to systematic differences in how the pathways are education persons on seizures? Curriculum guidance for courses is available [19]. Exact provision though, is at provider discretion. There may be an opportunity here for providers to share best practice.

Overall, our results suggest modification to pre-registration education alone may not be sufficient to address the sizeable number of AAs for seizures. Thus, other interventions should be considered. A range of macro, meso and micro factors potentially influence conveyance decisions [33] and work is underway to address some. One factor which has not been addressed is the minimal sharing of information between seizure specialists and emergency care providers. Consequently, clinicians in the out-of-hospital setting have limited access to information on the baseline health of the person they are seeing and referrals of patients to seizure specialists following contact with urgent emergency care providers remain patchy [4].

### Limitations

The sample for our, albeit cross-sectional, survey was large – representing ~ 10% of those studying in the UK to be a paramedic who were in year 2 or beyond at the time [34]. There was also minimal attrition. Limited evidence is available on the characteristics of student UK paramedics. Subject level data [34] at least indicates that the our sample was representative with regards sex. It is unknown, however, whether those studying via an apprenticeship were underrepresented.

Subsequent studies should consider using additional measures to assess perceived confidence to permit them to understand things in a more granular way. This is because the one we used, whilst comprising of questions on perceived knowledge of, ability to care for and confidence to care, only generates an overall confidence score. Also, it was not possible via the measures we used to directly compare and contrast confidence to care overall with confidence to specifically make a conveyance decision. Their scales were different.

## Conclusions

Student paramedics report relatively high perceived readiness to manage seizures, with a minority requesting further education. It is likely that enhancements to pre-registration education alone will not be sufficient to address the sizeable number of AAs for seizures.

### Supplementary Information

**Additional file 1.** Systematic literature search: Methods and summary of studies.

**Additional file 2.** Higher Education Institutions within UK that did and did not circulate invite and their characteristics.

**Additional file 3.** Full survey.

**Additional file 4.** Amendments made to items from Waltrich et al.’s perceived knowledge of, ability to care for and confidence to care questionnaire survey to make it suitable for use with trainee paramedics, rather than practicing paramedics.

## Data Availability

Anonymised data are available on reasonable request (from the corresponding author).

## Abbreviations

AA: Avoidable attendance
BCa: Bias-corrected and accelerated bootstrap
BSc: Bachelor of Science
ED: Emergency department
IQR: Interquartile range
M: Mean
MD: Mean difference
Mdn: Median
MSc: Master of Science
POMP: Percentage of maximum possible
SD: Standard deviation
UK: United Kingdom
