# Supplementary material for: Raring to go? A cross-sectional survey of student paramedics on how well they perceive their UK pre-registration course to be preparing them to manage suspected seizures
Source: BMC Emerg Med. 2023 Oct 8;23:119. doi: 10.1186/s12873-023-00889-5 (PMC10561511; doi:10.1186/s12873-023-00889-5)
Supplement: Supplementary file 1 — Additional file 1. Systematic literature search: Methods and summary of studies. [file 12873_2023_889_MOESM1_ESM.docx]

**additional file 1** Systematic literature search: Methods and summary of studies

**Methods**

*Identification*

Searches were made of PsycINFO, Medline and Scopus from inception until 10/10/2022. The following search terms (article titles, abstracts and keywords) were used and modified for the different databases:

1. pre-hospital OR paramedic OR ambulance
2. AND epilep* OR seizure OR convuls*
3. AND views OR experience OR training OR preparedness OR concerns OR readiness OR attitude OR behavi?ur OR knowledge OR belief

*Eligibility and screening*

To be eligible an article had to be reported in English, be published in a peer-reviewed journal and the study needed to have considered the views, experience, training, preparedness, concerns, readiness, attitudes, behaviour, knowledge or belief of paramedics on epilepsy or seizures in humans.

Titles/abstracts for identified articles were screened for eligibility by two reviewers (LA & HI), removing duplicates and obviously irrelevant studies. Full texts versions of the articles that they both agreed as ostensibly eligible were accessed and further reviewed for eligibility by both reviewers. Agreement between them was high (91%) and the one discrepancy was resolved through discussion.

**Results**

The selection process and reasons for exclusion are shown in the PRISMA flow chart below. Ultimately 8 eligible studies were identified. They are summarised in the Table. None of the studies involved trainee paramedics from the United Kingdom (UK).

**Figure** PRISMA Flow chart showing identification and selection assessment

Total identified (n = 259)

## Identification

PsychINFO
(n = 29)

Medline
(n = 89)

Scopus

(n=141)

Records after duplicates removed & screened

(n = 251)

Records excluded based on title and abstract
(n = 240)

## Eligibility

Full text articles examined

(n = 11)

Records excluded after full article review

(n =3)

Eligible studies identified

(n =8)

## Included

**Table** Summary of findings from eligible studies identified by systematic search

| **Authors** | **Country** | **Sample** | | | | **Method/s** | **Key findings** |
| --- | --- | --- | --- | --- | --- | --- | --- |
|  |  | ***Number of ambulance clinicians included*** | ***Did sample include any trainees?*** | ***What pre-registration training had participants typically completed?*** | ***Years participants qualified for*** | ***How was readiness for seizure management explored?*** | ***Pertinent to current study*** |
| Burrell et al. [1] | UK | 15 | No. | Vocational | Median 6 (IQR 2 to 11) | Semi-structured interview. | -Half said they lacked confidence making conveyance decisions.  - Self-resolved epileptic seizures Identified as particularly challenging.  - Insufficient training and guidance reported.  - Higher confidence ascribed to experience, not training. |
| Ernest et al. [2] | USA | Not specified. | Indirectly | Not specified | Not specified | A retrospective review of 71,683 cases seen by trainee paramedics during preceptorship. | - Identified frequency of different presentations to ambulance service by 1-16 age. Suggests this needs to be accounted for in preceptorship training to ensure it provides sufficient exposure to prepare clinicians.  - Seizures were less common in paediatric population with each year increase age. Most common in 0 to 2-year-old group. |
| Shah et al.[3] | USA | Not specified. | No | Not specified | Not specified | The medication management of 250 active seizing incidents (in in 1 to 18 year olds) by paramedics who had and had not received additional training (PediSTEPPs) was compared. The PediSTEPPs training was delivered as part of a non-randomised trial. | - No statistically significant difference between groups detected on the primary outcome measures of administration of dextrose for hypoglycaemia or midazolam for euglycemia. |
| Noble et al.[4] | UK | 19 | No | Vocational | Mean 20  (SD=9.6) | Semi-structured interview. | - Paramedics reported can often have limited confidence to manage seizures.  - A range of factors, including limited training, stated as influencing conveyance decisions beyond patient need and create a momentum for patients to be conveyed to emergency departments. |
| Sherratt et al.[5]* | UK | 19 | No | Vocational | Mean 20  (SD=9.6) | Semi-structured interview. | - Paramedics said seizure management had been ‘neglected’ within both pre- and post-registration training.  - They reported this often leads to low seizure management knowledge and confidence among paramedics. |
| Kinney et al.[6] | UK | 47 | No | Vocational | Mean 11.5 (range 2 to 27] | Structured questionnaire | - Paramedics rated their confidence for managing seizures. It was lowest for recognizing different seizure types (including non-epileptic attack disorder) and for making conveyance decisions.  -Training was not commonly cited as being key to any high confidence, rather experience and protocols were. |
| Carey et al.[7] | USA | 66 | No | Not specified | 10.6 (SD 5.6) | Semi-structured interview | - Paramedics, who had recently transported  actively seizing 0–17-year-old patients reported enablers and barriers to protocol adherent care for paediatric patients.  - Limited paediatric specific training was one of a range of system level barriers identified. Provider-level barriers were also identified. |
| Lammers et al.[8] | USA | 147 | No | Not specified | Median range across groups 4 to 7.2 | As part of a randomised controlled trial, the effect of 3 different educational interventions on paramedics ability to manage a range of paediatric presentations was assessed. Seizure was one of the presentations Outcome measure was a validated,  performance-based, simulated clinical assessment. | - Paediatric seizure management ability significantly improved compared to baseline for persons in each of the interventional educational groups.  - Percentage score improvement ranged from +11 to +15% across different educational groups.  - Ability did not significantly improve in the control group (score improvement +4.5%). |

*Notes:* IQR, interquartile range; PediSTEPPs, Pediatric Simulation Training for Emergency Prehospital Providers; UK, United Kingdom; US, United States of America; SD, standard deviation; * Study used same data set as Noble et al.. Sherratt et al. reported on at educational needs, whilst Noble et al. primarily reported seizure care experiences.

**REFERENCES**

1. Burrell, L., A. Noble, and L. Ridsdale, *Decision-making by ambulance clinicians in London when managing patients with epilepsy: a qualitative study.* Emergency Medicine Journal, 2013. **30**: p. 236–240.

2. Ernest, E.V., et al., *Prevalence of Unique Pediatric Pathologies Encountered by Paramedic Students Across Age Groups.* Prehospital and disaster medicine, 2016. **31**(4): p. 386–391.

3. Shah, M.I., et al., *Impact of High-Fidelity Pediatric Simulation on Paramedic Seizure Management.* Prehospital Emergency Care, 2016. **20**(4): p. 499–507.

4. Noble, A., et al., *Qualitative study of paramedics’ experiences of managing seizures: a national perspective from England.* BMJ Open, 2016. **6**: p. e014022.

5. Sherratt, F., et al., *Paramedics’ views on their seizure management learning needs: a qualitative study in England.* BMJ Open, 2017. **7**: p. e014024.

6. Kinney, M.O., S.J. Hunt, and C. McKenna, *A self-completed questionnaire study of attitudes and perceptions of paramedic and prehospital practitioners towards acute seizure care in Northern Ireland.* Epilepsy & Behavior, 2018. **81**: p. 115–118.

7. Carey, J.M., et al., *Paramedic-Identified Enablers of and Barriers to Pediatric Seizure Management: A Multicenter, Qualitative Study.* Prehospital Emergency Care, 2019. **23**(6): p. 870-881.

8. Lammers, R.L., et al., *Comparison of Four Methods of Paramedic Continuing Education in the Management of Pediatric Emergencies.* Prehospital Emergency Care, 2022. **26**(4): p. 463–475.
