# Supplementary material for: Raring to go? A cross-sectional survey of student paramedics on how well they perceive their UK pre-registration course to be preparing them to manage suspected seizures
Source: BMC Emerg Med. 2023 Oct 8;23:119. doi: 10.1186/s12873-023-00889-5 (PMC10561511; doi:10.1186/s12873-023-00889-5)
Supplement: Supplementary file 2 — Additional file 2. Higher Education Institutions within UK that did and did not circulate invite and their characteristics. [file 12873_2023_889_MOESM2_ESM.docx]

**Additional File 2** Higher Education Institutions within UK that did and did not circulate invite and their characteristics

|  | **Was advert circulated at HEI site?** | | | | | | | |
| --- | --- | --- | --- | --- | --- | --- | --- | --- |
|  | ***Yes*** | | | | ***No*** | | | |
|  | ***Provider name*** | ***Country*** | ***Number of years eligible to participate across approved courses offered*** | ***Provider name*** | | ***Country*** | ***Number of years eligible to participate across approved courses offered*** |  |
|  | Birmingham City University | England | Two (Years 2, 3) | Anglia Ruskin University | | England | Two (Years 2, 3) |  |
|  | Bournemouth University | England | Two (Years 2, 3) | Canterbury Christ Church University | | England | Two (Years 2, 3) |  |
|  | Buckinghamshire New University | England | One (Year 2) | Coventry University | | England | Two (Years 2, 3) |  |
|  | De Montfort University | England | Two (Years 2, 3) | Keele University | | England | One (Year 2) |  |
|  | Edge Hill University | England | Three (Years 2, 3, 4) | Sheffield Hallam University | | England | Two (Years 2, 3) |  |
|  | Liverpool John Moores University | England | Two (Years 2, 3) | St George's, University of London | | England | Two (Years 2, 3) |  |
|  | Nottingham Trent University | England | Two (Years 2, 3) | Staffordshire University | | England | Two (Years 2, 3) |  |
|  | Oxford Brookes University | England | Two (Years 2, 3) | The University of Northampton | | England | Two (Years 2, 3) |  |
|  | Teesside University | England | Two (Years 2, 3) | University Huddersfield | | England | One (Year 2) |  |
|  | The University of Bolton | England | One (Year 2) | University of Bradford | | England | Two (Years 2, 3) |  |
|  | University of Bedfordshire | England | Two (Years 2, 3) | University of Gloucestershire | | England | Two (Years 2, 3) |  |
|  | University of Brighton | England | Two (Years 2, 3) | University of Hull | | England | Two (Years 2, 3) |  |
|  | University of Central Lancashire | England | Two (Years 2, 3) | University of Suffolk | | England | Two (Years 2, 3) |  |
|  | University of Cumbria | England | Two (Years 2, 3) | Queen Margaret University | | Scotland | Two (Years 2, 3) |  |
|  | University of East Anglia | England | Two (Years 2, 3) | University of the West of Scotland | | Scotland | Two (Years 2, 3) |  |
|  | University of Greenwich | England | Two (Years 2, 3) |  | |  |  |  |
|  | University of Hertfordshire | England | Two (Years 2, 3) |  | |  |  |  |
|  | University of Lincoln | England | Two (Years 2, 3) |  | |  |  |  |
|  | University of Plymouth | England | Two (Years 2, 3) |  | |  |  |  |
|  | University of Portsmouth | England | Two (Years 2, 3) |  | |  |  |  |
|  | University of Sunderland | England | Two (Years 2, 3) |  | |  |  |  |
|  | University of Surrey | England | Two (Years 2, 3) |  | |  |  |  |
|  | University of the West of England | England | Two (Years 2, 3) |  | |  |  |  |
|  | University of West London | England | Two (Years 2, 3) |  | |  |  |  |
|  | University of Wolverhampton | England | Two (Years 2, 3) |  | |  |  |  |
|  | University of Worcester | England | Two (Years 2, 3) |  | |  |  |  |
|  | University of Ulster | N. Ireland | One (Year 2) |  | |  |  |  |
|  | Glasgow Caledonian University | Scotland | Two (Years 2, 3) |  | |  |  |  |
|  | Robert Gordon University | Scotland | Two (Years 2, 3) |  | |  |  |  |
|  | University of Stirling | Scotland | Two (Years 2, 3) |  | |  |  |  |
|  | Swansea University | Wales | Two (Years 2, 3) |  | |  |  |  |
|  | | England: 26 (83.9%)  Scotland: 3 (9.7%)  Wales: 1 (3.2%)  N. Ireland: 1 (3.2%) | One: 3 (9.7%)  Two: 27 (87.1%)  Three: 1 (3.2%) |  | | England: 13 (86.7%)  Scotland: 2 (13.3)  Wales: 0  N. Ireland: 0 | One: 2 (13.3%)  Two: 13 (86.7%)  Three: 0 |  |

*Notes* HEI, Higher Education Institute; N., Northern
