# Supplementary material for: Raring to go? A cross-sectional survey of student paramedics on how well they perceive their UK pre-registration course to be preparing them to manage suspected seizures
Source: BMC Emerg Med. 2023 Oct 8;23:119. doi: 10.1186/s12873-023-00889-5 (PMC10561511; doi:10.1186/s12873-023-00889-5)
Supplement: Supplementary file 3 — Additional file 3. Full survey. [file 12873_2023_889_MOESM3_ESM.docx]

**Additional File 3** Full survey*

* Note that the actual order in which presentation were asked about was randomised.

Start of Block: Landing page with welcome, logo and embedded link for information sheet

 
As the next generation of paramedics, it is important to understand what can be done to help trainees feel ready for practice. University of Liverpool and Liverpool John Moores University are therefore doing a survey.
 
It only takes 5 to 10 minutes. The first 300 people submitting a complete response each get a £5 voucher.
 
The survey asks trainees how they feel programmes are preparing them and if any changes are required.
 You can take part if you:

 • Are aged 16 years or over.
 • Are enrolled on an HCPC approved paramedic training programme.
• Are able to complete a survey in English by yourself
 • Do not have a terminal illness or severe psychiatric condition
 • Live in the UK
 • People in all years of study can take part (except those in year 1, sorry!).
 A participant information form, with more details about the study, can be found [HERE](https://livpsych.eu.qualtrics.com/CP/File.php?F=F_bmFlHNoEqbTrd5k)

 If any help is required while completing the study, please email us at sz.res.group@liverpool.ac.uk

 This study has been reviewed and approved by the University of Liverpool Research Ethics Committee (REF: 11962).

| 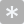 |
| --- |

**To take part in the study you need to agree to the following things:**

- I confirm that I have read the information sheet dated 28.10.22 (v1.0) for the above study. (1)
- I confirm that I am suitable to take part in the study. (2)
- I have had the opportunity to consider the information and any questions I had have been answered satisfactorily. (3)
- I understand that my participation is voluntary. I am free to withdraw at any time without giving any reason, and that this will not affect my studies or training. (4)
- I have the ability to decide for myself whether I do or do not want to take part in the study. (5)
- I agree to take part in the above study. (6)

End of Block: Landing page with welcome, logo and embedded link for information sheet

Start of Block: Participant characteristics

**First, we would like to know a bit about you.**

**Please tell us your current age in years.**
 I am... (select the option that applies)

▼ 16 (1) ... 80 and above (65)

**What is your sex?** (This might be different to your gender identity)

- Male (1)
- Female (2)
- Prefer not to say (3)

**How are you currently training to be a paramedic?**
 I am... (select the option that applies)

- On an approved course in paramedic science at a university (1)
- Completing a degree level apprenticeship in paramedic science with an ambulance service (2)
- Other - please describe: (3) __________________________________________________

**Have you ever worked as an Emergency Medical Technician (EMT)** (either as part of your current training or as a profession before starting it?)

- Yes (1)
- No (2)

| 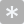 |
| --- |

**What year of training are you currently in?**
 I am currently in... (select the option that applies)

- Year 1 (1)
- Year 2 (2)
- Year 3 (3)
- Year 4 (4)

**In which country are you training in?**

- England (1)
- Wales (2)
- Scotland (3)
- Northern Ireland (4)

Display This Question:

If In which country are you training in? = England

**In which English region are you mostly training in?**

- Yorkshire and the Humber (e.g., Leeds, etc.) (1)
- West Midlands (e.g., Birmingham, etc.) (2)
- South West (e.g., Taunton, etc.) (3)
- South East (e.g., Guildford, etc.) (4)
- North West (e.g., Liverpool, Manchester, etc.) (5)
- London/Greater London (6)
- East of England (e.g., Flempton, etc.) (7)
- East Midlands (e.g., Nottingham, etc.) (8)

End of Block: Participant characteristics

Start of Block: Knowledge

**We would now like to ask you about your sense of preparedness to manage three different presentations.**
 Firstly, we would like to ask you about your knowledge of each of them.

End of Block: Knowledge

Start of Block: Knowledge of seizure presentations

**The presentation we are focusing on here is seizures.**
 
**Please tell us how much you agree or disagree with each of the following statements** (select your choices from the drop-down menus):
 
**My knowledge of seizure patients is comprehensive.**

- 1 = Strongly disagree (1)
- 2 = Disagree (2)
- 3 = Neutral (3)
- 4 = Agree (4)
- 5 = Strongly agree (5)

**I am able to recognise different types of seizure presentations.**

- 1 = Strongly disagree (1)
- 2 = Disagree (2)
- 3 = Neutral (3)
- 4 = Agree (4)
- 5 = Strongly agree (5)

**I am knowledgeable on how to assess and treat a patient presenting with a seizure problem in the prehospital setting.**

- 1 = Strongly disagree (1)
- 2 = Disagree (2)
- 3 = Neutral (3)
- 4 = Agree (4)
- 5 = Strongly agree (5)

**My knowledge of the different types of seizure presentations is poor.**

- 1 = Strongly disagree (1)
- 2 = Disagree (2)
- 3 = Neutral (3)
- 4 = Agree (4)
- 5 = Strongly agree (5)

**I am easily able to recognise when a patient in presenting with a seizure problem.**

- 1 = Strongly disagree (1)
- 2 = Disagree (2)
- 3 = Neutral (3)
- 4 = Agree (4)
- 5 = Strongly agree (5)

End of Block: Knowledge of seizure presentations

Start of Block: Knowledge of breathing problem presentations

**The presentation we are focusing on here is breathing problems.**
 
**Please tell us how much you agree or disagree with each of the following statements** (select your choices from the drop-down menus):
 
 
**My knowledge of breathing problem patients is comprehensive.**

- 1 = Strongly disagree (1)
- 2 = Disagree (2)
- 3 = Neutral (3)
- 4 = Agree (4)
- 5 = Strongly agree (5)

**I am able to recognise different types of breathing problems presentations.**

- 1 = Strongly disagree (1)
- 2 = Disagree (2)
- 3 = Neutral (3)
- 4 = Agree (4)
- 5 = Strongly agree (5)

**I am knowledgeable on how to assess and treat a patient presenting with a breathing problem in the prehospital setting.**

- 1 = Strongly disagree (1)
- 2 = Disagree (2)
- 3 = Neutral (3)
- 4 = Agree (4)
- 5 = Strongly agree (5)

**My knowledge of the different types of breathing problem presentations is poor.**

- 1 = Strongly disagree (1)
- 2 = Disagree (2)
- 3 = Neutral (3)
- 4 = Agree (4)
- 5 = Strongly agree (5)

**I am easily able to recognise when a patient is presenting with a breathing problem.**

- 1 = Strongly disagree (1)
- 2 = Disagree (2)
- 3 = Neutral (3)
- 4 = Agree (4)
- 5 = Strongly agree (5)

End of Block: Knowledge of breathing problem presentations

Start of Block: Knowledge of headache presentations

**The presentation we are focusing on here is headaches.**
 
**Please tell us how much you agree or disagree with each of the following statements** (select your choices from the drop-down menus):
 **My knowledge of headache patients is comprehensive.**

- 1 = Strongly disagree (1)
- 2 = Disagree (2)
- 3 = Neutral (3)
- 4 = Agree (4)
- 5 = Strongly agree (5)

**I am able to recognise different types of headache presentations.**

- 1 = Strongly disagree (1)
- 2 = Disagree (2)
- 3 = Neutral (3)
- 4 = Agree (4)
- 5 = Strongly agree (5)

**I am knowledgeable on how to assess and treat a patient presenting with a headache problem in the prehospital setting.**

- 1 = Strongly disagree (1)
- 2 = Disagree (2)
- 3 = Neutral (3)
- 4 = Agree (4)
- 5 = Strongly agree (5)

**My knowledge of the different types of headache presentations is poor.**

- 1 = Strongly disagree (1)
- 2 = Disagree (2)
- 3 = Neutral (3)
- 4 = Agree (4)
- 5 = Strongly agree (5)

**I am easily able to recognise when a patient in presenting with a headache problem.**

- 1 = Strongly disagree (1)
- 2 = Disagree (2)
- 3 = Neutral (3)
- 4 = Agree (4)
- 5 = Strongly agree (5)

End of Block: Knowledge of headache presentations

Start of Block: Perceived ability to provide care

**We would now like to ask you about your perceived ability to provide care for the different presentations.**

 **Please tell us how much you agree or disagree with each of the following statements** (select your choices from the drop down menus):

End of Block: Perceived ability to provide care

Start of Block: Perceived ability to provide care for seizure presentations

**The presentation we are focusing on here is seizures.**
 
 
**I believe my training is preparing me well to provide care that helps seizure patients.**

- 1 = Strongly disagree (1)
- 2 = Disagree (2)
- 3 = Neutral (3)
- 4 = Agree (4)
- 5 = Strongly agree (5)

**If I were to attend to a seizure patient, I know how to provide management/treatment that will assist the patient's condition.**

- 1 = Strongly disagree (1)
- 2 = Disagree (2)
- 3 = Neutral (3)
- 4 = Agree (4)
- 5 = Strongly agree (5)

**I believe my education and training is preparing me well to provide care that benefits seizure patients.**

- 1 = Strongly disagree (1)
- 2 = Disagree (2)
- 3 = Neutral (3)
- 4 = Agree (4)
- 5 = Strongly agree (5)

End of Block: Perceived ability to provide care for seizure presentations

Start of Block: Perceived ability to provide care for breathing problem presentations

**The presentation we are focusing on here is breathing problems.**

 **I believe my training is preparing me well to provide care that helps breathing problem patients.**

- 1 = Strongly disagree (1)
- 2 = Disagree (2)
- 3 = Neutral (3)
- 4 = Agree (4)
- 5 = Strongly agree (5)

**If I were to attend to a breathing problem patient, I know how to provide management/treatment that will assist the patient's condition.**

- 1 = Strongly disagree (1)
- 2 = Disagree (2)
- 3 = Neutral (3)
- 4 = Agree (4)
- 5 = Strongly agree (5)

**I believe my education and training is preparing me well to provide care that benefits breathing problem patients.**

- 1 = Strongly disagree (1)
- 2 = Disagree (2)
- 3 = Neutral (3)
- 4 = Agree (4)
- 5 = Strongly agree (5)

End of Block: Perceived ability to provide care for breathing problem presentations

Start of Block: Perceived ability to provide care for headache presentations

**The presentation we are focusing on here is headaches.**

 **I believe my training is preparing me well to provide care that helps headache patients.**

- 1 = Strongly disagree (1)
- 2 = Disagree (2)
- 3 = Neutral (3)
- 4 = Agree (4)
- 5 = Strongly agree (5)

**If I were to attend to a headache patient, I know how to provide management/treatment that will assist the patient's condition.**

- 1 = Strongly disagree (1)
- 2 = Disagree (2)
- 3 = Neutral (3)
- 4 = Agree (4)
- 5 = Strongly agree (5)

**I believe my education and training is preparing me well to provide care that benefits headache patients.**

- 1 = Strongly disagree (1)
- 2 = Disagree (2)
- 3 = Neutral (3)
- 4 = Agree (4)
- 5 = Strongly agree (5)

End of Block: Perceived ability to provide care for headache presentations

Start of Block: Confidence

**Finally, we would like you to tell us how confident you think you would be in managing the presentations.**

 **Please tell us how much you agree or disagree with each of the following statements** (select your choices from the drop down menus):

End of Block: Confidence

Start of Block: Confidence towards seizure presentations

**The presentation we are focusing on here is seizures.**
 
**I am, or believe I would be, very confident when attending a patient presenting with a seizure problem.**

- 1 = Strongly disagree (1)
- 2 = Disagree (2)
- 3 = Neutral (3)
- 4 = Agree (4)
- 5 = Strongly agree (5)

**I do, or believe I would, feel anxious when attending a patient presenting with a seizure problem.**

- 1 = Strongly disagree (1)
- 2 = Disagree (2)
- 3 = Neutral (3)
- 4 = Agree (4)
- 5 = Strongly agree (5)

**I do, or believe I would, feel stressed when called to a patient presenting with a seizure problem.**

- 1 = Strongly disagree (1)
- 2 = Disagree (2)
- 3 = Neutral (3)
- 4 = Agree (4)
- 5 = Strongly agree (5)

**I feel confident that I can/could assess and treat a patient with a seizure problem to a high standard.**

- 1 = Strongly disagree (1)
- 2 = Disagree (2)
- 3 = Neutral (3)
- 4 = Agree (4)
- 5 = Strongly agree (5)

**I would feel comfortable in my ability if I were to attend a patient with a seizure problem.**

- 1 = Strongly disagree (1)
- 2 = Disagree (2)
- 3 = Neutral (3)
- 4 = Agree (4)
- 5 = Strongly agree (5)

End of Block: Confidence towards seizure presentations

Start of Block: Confidence towards breathing problem presentations

**The presentation we are focusing on here is breathing problems.**

 **I am, or believe I would be, very confident when attending a patient presenting with a breathing problem.**

- 1 = Strongly disagree (1)
- 2 = Disagree (2)
- 3 = Neutral (3)
- 4 = Agree (4)
- 5 = Strongly agree (5)

**I do, or believe I would, feel anxious when attending a patient presenting with a breathing problem.**

- 1 = Strongly disagree (1)
- 2 = Disagree (2)
- 3 = Neutral (3)
- 4 = Agree (4)
- 5 = Strongly agree (5)

**I do, or believe I would, feel stressed when called to a patient presenting with a breathing problem.**

- 1 = Strongly disagree (1)
- 2 = Disagree (2)
- 3 = Neutral (3)
- 4 = Agree (4)
- 5 = Strongly agree (5)

**I feel confident that I can/could assess and treat a patient with a breathing problem to a high standard.**

- 1 = Strongly disagree (1)
- 2 = Disagree (2)
- 3 = Neutral (3)
- 4 = Agree (4)
- 5 = Strongly agree (5)

**I would feel comfortable in my ability if I were to attend a patient with a breathing problem.**

- 1 = Strongly disagree (1)
- 2 = Disagree (2)
- 3 = Neutral (3)
- 4 = Agree (4)
- 5 = Strongly agree (5)

End of Block: Confidence towards breathing problem presentations

Start of Block: Confidence towards headache presentations

**The presentation we are focusing on here is headaches.**

 **I am, or believe I would be, very confident when attending a patient presenting with a headache problem.**

- 1 = Strongly disagree (1)
- 2 = Disagree (2)
- 3 = Neutral (3)
- 4 = Agree (4)
- 5 = Strongly agree (5)

**I do, or believe I would, feel anxious when attending a patient presenting with a headache problem.**

- 1 = Strongly disagree (1)
- 2 = Disagree (2)
- 3 = Neutral (3)
- 4 = Agree (4)
- 5 = Strongly agree (5)

**I do, or believe I would, feel stressed when called to a patient presenting with a headache problem.**

- 1 = Strongly disagree (1)
- 2 = Disagree (2)
- 3 = Neutral (3)
- 4 = Agree (4)
- 5 = Strongly agree (5)

**I feel confident that I can/could assess and treat a patient with a headache problem to a high standard.**

- 1 = Strongly disagree (1)
- 2 = Disagree (2)
- 3 = Neutral (3)
- 4 = Agree (4)
- 5 = Strongly agree (5)

**I would feel comfortable in my ability if I were to attend a patient with a headache problem.**

- 1 = Strongly disagree (1)
- 2 = Disagree (2)
- 3 = Neutral (3)
- 4 = Agree (4)
- 5 = Strongly agree (5)

End of Block: Confidence towards headache presentations

Start of Block: Conveyance confidence

**We would now like to ask you how confident you would say you would be in deciding whether or not to take people with the presentations to a hospital emergency department (ED)** (select your choices from the drop-down menus):

End of Block: Conveyance confidence

Start of Block: Conveyance confidence for seizure presentations

**How confident would you say you would be in deciding whether or not to convey a seizure patient to ED?**

- 1 = Not at all confident (1)
- 2 = Slightly confident (2)
- 3 = Reasonably confident (3)
- 4 = Very confident (4)
- 5 = Extremely confident (5)

End of Block: Conveyance confidence for seizure presentations

Start of Block: Conveyance confidence for breathing problem presentations

**How confident would you say you would be in deciding whether or not to convey a breathing problem patient to ED?**

- 1 = Not at all confident (1)
- 2 = Slightly confident (2)
- 3 = Reasonably confident (3)
- 4 = Very confident (4)
- 5 = Extremely confident (5)

End of Block: Conveyance confidence for breathing problem presentations

Start of Block: Conveyance confidence for headache presentations

**How confident would you say you would be in deciding whether or not to convey a headache patient to ED?**

- 1 = Not at all confident (1)
- 2 = Slightly confident (2)
- 3 = Reasonably confident (3)
- 4 = Very confident (4)
- 5 = Extremely confident (5)

End of Block: Conveyance confidence for headache presentations

Start of Block: Pre-registration opinions

**We would like to know whether you think your pre-registration should be giving more attention to the different presentations?**

End of Block: Pre-registration opinions

Start of Block: Pre-registration opinions on seizure presentations

**Do you think you should/should have received more training on seizures via your pre-registration programme?**

- Yes (1)
- No (2)
- Unsure (3)

Display This Question:

If Do you think you should/should have received more training on seizures via your pre-registration... = Yes

**To create space for this, what do you think might be removed or there could be less of?**

________________________________________________________________

End of Block: Pre-registration opinions on seizure presentations

Start of Block: Pre-registration opinions on breathing problem presentations

**Do you think you should/should have received more training on breathing problems via your pre-registration programme?**

- Yes (1)
- No (2)
- Unsure (3)

Display This Question:

If Do you think you should/should have received more training on breathing problems via your pre-reg... = Yes

**To create space for this, what do you think might be removed or there could be less of?**

________________________________________________________________

End of Block: Pre-registration opinions on breathing problem presentations

Start of Block: Pre-registration opinions on headache presentations

**Do you think you should/should have received more training on headaches via your pre-registration programme?**

- Yes (1)
- No (2)
- Unsure (3)

Display This Question:

If Do you think you should/should have received more training on headaches via your pre-registration... = Yes

**To create space for this, what do you think might be removed or there could be less of?**

________________________________________________________________

End of Block: Pre-registration opinions on headache presentations

Start of Block: Experience of sustainable health care education and views on the role of the NHS

**This is the final question of the survey.**

 **Safely reducing the number of clinically unnecessary journeys to ED by ambulance is an NHS target. One possible consequence of fewer clinically unnecessary ambulance journeys to EDs would be fewer carbon emissions.**

**How important do you think it is for the health and care system to work in a way that supports the environment, such as improving resource efficiency, reducing carbon emissions and reducing waste?**

- 1 = Strongly agree (1)
- 2 = Agree to some extent (2)
- 3 = Disagree to some extent (3)
- 4 = Strongly disagree (4)
- 5 = Don't know (5)

End of Block: Experience of sustainable health care education and views on the role of the NHS

Start of Block: End of the survey

**You have now reached the end of the survey.**

**Shopping voucher:**

 The first 300 people who submit complete responses to the survey will each receive a £5 shopping voucher. If you would like to be considered for this, please enter you name and email address below:

- Your name: (1) __________________________________________________
- Your email address: (2) __________________________________________________

**Some potentially helpful resources:**

 If you would like to know more about the 3 presentations focused upon by this survey we would direct you to: The College of Paramedic’s E-Learning page (https://collegeofparamedics.co.uk/COP/ProfessionalDevelopment/E-Learning.aspx) The Joint Royal Colleges Ambulance Liaison Committee (JRCALC) guidelines (https://aace.org.uk/clinical-practice-guidelines/).
 Whilst not directly related to the RISE survey, we are aware that studying can at times be challenging. We would therefore like to take the opportunity to highlight the following resources in case these are of interest: https://bluelighttogether.org.uk/ambulance/coping-with-student-life-for-trainee-paramedics/ https://www.nhs.uk/mental-health/children-and-young-adults/help-for-teenagers-young-adults-and-students/student-stress-self-help-tips/ https://collegeofparamedics.co.uk/COP/Member_/Paramedic_Mental_Health_and_Wellbeing.aspx

**Thank you for submitting your answers. If you need to speak to someone about the study, you can contact the research team by emailing** [sz.res.group@liverpool.ac.uk](mailto:sz.res.group@liverpool.ac.uk)

End of Block: End of the survey
