# Supplementary material for: Raring to go? A cross-sectional survey of student paramedics on how well they perceive their UK pre-registration course to be preparing them to manage suspected seizures
Source: BMC Emerg Med. 2023 Oct 8;23:119. doi: 10.1186/s12873-023-00889-5 (PMC10561511; doi:10.1186/s12873-023-00889-5)
Supplement: Supplementary file 4 — Additional file 4. Amendments made to items from Waltrich et al.’s perceived knowledge of, ability to care for and confidence to care questionnaire survey to make it suitable for use with trainee paramedics, rather than practicing paramedics. [file 12873_2023_889_MOESM4_ESM.docx]

**ADDITIONAL FILE 4** Amendments made to items from Waltrich et al.’s perceived knowledge of, ability to care for and confidence to care questionnaire survey to make it suitable for use with trainee paramedics, rather than practicing paramedics

| **Subscale** | **Item as used within current study** | **Original item as used by Waltrich et al.** |
| --- | --- | --- |
| **Knowledge of condition:** |  |  |
| **1** | ‘My knowledge X patients is comprehensive | No change |
| **2** | ‘I am able to recognise different types of X presentations | No change |
| **3** | ‘I am knowledgeable on how to assess and treat a patient presenting with a X problem in the prehospital setting’ | No change |
| **4** | ‘My knowledge of the different types of X presentations is poor’ (negatively coded) | No change |
| **5** | ‘I am easily able to recognise when a patient is presenting with a X problem’ | No change |
| **Ability to care:** |  |  |
| **1** | ‘I believe my training is preparing me well to provide care that helps X patients’ | ‘I believe I am able to provide care that helps X patients’ |
| **2** | ‘If I were to attend to a X patient, I know how to provide management/treatment that will assist the patient’s condition’ | ‘When attending a X patient, I know how to provide management/treatment that will assist the patient’s condition’ |
| **3** | ‘I believe my education and training is preparing me well to provide care that benefits X patients’ | ‘I believe my education and training enables me to provide care that benefits X patients’ |
| **4** | - | Excluded item: ‘The care I provide to X patients provides benefit and improves their condition’ |
| **5** | - | Excluded item: ‘The care I can provide X patients is limited and rarely beneficial for the patient’ (negatively coded) |
| **Confidence to care:** |  |  |
| **1** | ‘I am, or believe I would be, very confident when attending a patient presenting with a X problem’. | ‘I am very confident when attending a patient presenting with a X problem’. |
| **2** | ‘I do, or believe I would, feel anxious when attending a patient presenting with a X problem’ (negatively coded) | ‘I feel anxious when attending a patient presenting with a X problem’ (negatively coded) |
| **3** | ‘I do, or believe I would, feel stressed when called to a patient presenting with a X problem’ (negatively coded) | ‘I feel stressed when called to a patient presenting with a X problem’ (negatively coded) |
| **4** | ‘I feel confident that I can/could assess and treat a patient with a X problem to a high standard’ | ‘I feel stressed when called to a patient presenting with a X problem’ (negatively coded) |
| **5** | ‘I would feel comfortable in my ability if I were to attend a patient with a X problem’ | ‘I feel comfortable in my ability when attending a patient with a X health problem’ |
